# Seroprevalence and Determinants of Hepatitis B and C Viral Infections Among Pregnant Women Attending Debark General Hospital, Northwest Ethiopia

**DOI:** 10.1155/bmri/6510690

**Published:** 2026-02-23

**Authors:** Gebre Ayanaw Alula, Shegasew Tesema

**Affiliations:** ^1^ Department of Biology, Debark University, Debark, Ethiopia, dku.edu.et; ^2^ Department of Sport Science, Debark University, Debark, Ethiopia, dku.edu.et

**Keywords:** Ethiopia, hepatitis B, hepatitis C, pregnant women, risk factors, seroprevalence

## Abstract

Viral hepatitis, caused by the hepatitis B virus (HBV) and the hepatitis C virus (HCV), remains a major public health concern among pregnant women due to the risk of vertical transmission and severe maternal and neonatal complications. HBV and HCV infections are characterized by significant morbidity and mortality. This study was aimed at determining the seroprevalence and identifying determinants of HBV, HCV, and HBV‐HCV coinfection among pregnant women attending antenatal care at Debark General Hospital, Northwest Ethiopia. Data on sociodemographic characteristics and potential risk factors were collected using a structured questionnaire. Five milliliters of venous blood were obtained from each participant to detect HBV antigens and HCV antibodies. Statistical analysis was carried out using descriptive and logistic regression models. Descriptive statistics summarized the findings, and logistic regression identified independent infection predictors. Variables with *p* ≤ 0.25 in univariate analysis entered the multivariate model, and *p* < 0.05 was considered statistically significant. The total seroprevalence of HBV infection was 5.9%, HCV infection 2.3%, and coinfection with HBV‐HCV 1.2%. HBV infection was significantly associated with a history of sharp object injuries, contact with a person infected with HBV, and prior receipt of blood transfusions. HCV infection was significantly associated with sharp object injuries, having multiple sexual partners, and a history of blood transfusion, while HBV‐HCV coinfection was associated with multiple sexual partnerships and contact with infected individuals. These results indicate that HBV and HCV remain major public health concerns among pregnant women in Debark. To reduce maternal and neonatal morbidity, routine antenatal screening, expanded coverage of HBV vaccination, adherence to safe medical and injection practices, and targeted evidence‐based health education are recommended.

## 1. Introduction

Hepatitis B virus (HBV) and hepatitis C virus (HCV) infections represent significant global public health concerns due to their high prevalence, frequent progression to chronic disease, and link to serious long‐term complications, such as cirrhosis, end‐stage liver disease, and hepatocellular carcinoma [1, 2]. Worldwide, an estimated 254 million people live with chronic HBV infection, and approximately 71 million have chronic HCV infection, together contributing markedly to liver‐related illness and death each year [3, 4]. The epidemiological impact of these infections is unevenly distributed, with a particularly heavy burden in low‐ and middle‐income countries, especially in sub‐Saharan Africa, where prevalence is high and access to preventive measures, diagnostic tools, and antiviral treatment remains insufficient [5].

Across sub‐Saharan Africa, a growing body of data continues to highlight the public health importance of viral hepatitis among pregnant women. A recent facility‐based study in Malawi found that about 6% of pregnant women attending antenatal clinics were infected with HBV, while the prevalence of HCV infection was approximately 1.2%, as noted by Nkhata et al. [6]. The investigation further identified key methodological constraints linked to the predominant reliance on rapid diagnostic tests instead of more sensitive laboratory‐based assays, underscoring the need to broaden screening efforts and improve diagnostic precision in order to more accurately define the burden and transmission patterns of HBV and HCV in pregnant women in this setting.

Pregnant women represent a particularly at‐risk group due to the possibility of vertical transmission, which is a key route for maintaining ongoing infection. Without timely prophylactic measures, babies born to mothers infected with HBV have an estimated 80%–90% chance of developing chronic infection [7, 8]. Although vertical transmission of HCV is less common—occurring in approximately 4%–10% of exposed pregnancies—it can result in persistent infection in the newborn and thus contribute to long‐term morbidity [9]. Furthermore, maternal HBV or HCV infection has been linked to unfavorable obstetric and perinatal outcomes, including preterm birth, low birth weight, and higher perinatal mortality [10].

HBV and HCV share similar modes of transmission, including parenteral exposure through unsafe injection practices, transfusion of infected blood or blood products, invasive surgical procedures, and sexual and vertical (mother‐to‐child) transmission routes. While effective HBV vaccines and antiviral therapies are available to reduce the risk of perinatal transmission, routine screening of pregnant women and universal provision of a timely birth dose vaccine are not consistently implemented in many Ethiopian healthcare settings. Currently, no prophylactic vaccine exists for HCV, and treatment options during pregnancy remain limited, making robust prevention strategies more challenging [11].

In Ethiopia, the reported prevalence of HBV among pregnant women ranges from 3% to 7.8%, whereas HCV prevalence ranges from 0.5% to 5%, reflecting substantial geographic variation [12, 13]. However, most existing studies have been restricted to specific health facilities or limited geographic areas, leaving major gaps in national epidemiological evidence. This gap is particularly pronounced in northern Ethiopia, where comprehensive and high‐quality data on the prevalence and determinants—factors that increase the likelihood of infection, such as exposure to unsafe medical practices, previous contact with infected individuals, or behavioral risk factors—of HBV and HCV infection among pregnant women are scarce [14, 15]. These knowledge gaps hinder the development of effective antenatal screening policies, vaccination programs, and targeted interventions to prevent vertical transmission of viral hepatitis.

Given this context, this study was aimed at assessing the seroprevalence and associated risk factors (determinants) for HBV and HCV infections among pregnant women attending antenatal care at Debark General Hospital in northwest Ethiopia. The findings are expected to provide evidence to inform health policy, improve antenatal care services, and guide the design of context‐appropriate preventive strategies to reduce the burden of viral hepatitis in this population.

## 2. Methodology

### 2.1. Study Area

The research was carried out at Debark General Hospital, located in Debark town in the North Gondar Zone of Ethiopia, about 830 km from Addis Ababa (13°8 ^′^ N, 37°54 ^′^ E; elevation 2850 m above sea level). The region experiences an average annual rainfall between 1900 and 2400 mm, with ambient temperatures ranging from 12°C to 20°C (North Gondar Zone Administration Office, 2018). The largely rural community depends mainly on mixed agriculture for its livelihood, cultivating key crops such as wheat, barley, peas, beans, and chickpeas (North Gondar Zone Agricultural and Rural Development Office, 2019). Based on the 2007 national census, the North Gondar Zone had a total population of 905,680, of which 425,846 were men and 479,834 were women. The healthcare in the area is provided primarily by one general hospital and three primary hospitals serving the neighboring populations.

### 2.2. Study Design and Period

A hospital‐based cross‐sectional study was carried out from January to June 2024 to determine seroprevalence and explore factors linked to HBV and HCV infections among pregnant women receiving antenatal care at Debark General Hospital.

### 2.3. Source and Study Population

The source population included all pregnant women who received antenatal care services in the hospital. The study population consisted of those who voluntarily gave their informed consent to participate in the study, which required providing a venous blood sample and completing a structured questionnaire on potential risk factors.

### 2.4. Inclusion and Exclusion Criteria

Pregnant women at any stage of gestation who provided their written informed consent were considered eligible for participation. Exclusion criteria included a previous history of HBV vaccination, use of antiviral therapy for HCV infection within the last 2 weeks, and the presence of serious medical conditions that prevented participation in or completion of the study questionnaire.

### 2.5. Sample Size and Sampling Technique

The sample size was calculated using the single population proportion formula, *n* = *Z*2*p* (1 − *p*)/*d*2. For this calculation, a prevalence of HBV (*p*) of 5.8% reported from Bahir Dar [16] was used, assuming a 95% confidence level and a 3% margin of error (*d*), with an additional 10% added to account for potential nonresponse. Based on these parameters, the final sample size was 256 participants. Subsequently, study subjects were chosen using a systematic random sampling method.

### 2.6. Data Collection Methods

#### 2.6.1. Questionnaire Survey

A structured questionnaire, previously pilot tested on a small group of pregnant women, was used to collect information on sociodemographic characteristics (e.g., age, residence, education, marital status, and occupation) and potential risk factors for HBV and HCV infections (e.g., history of blood transfusion, sharp object injuries, multiple sexual partners, contact with infected individuals, and other behavioral or clinical exposures).

The objectives of the study were clearly explained to all eligible participants, and written informed consent was obtained prior to data collection. Literate women completed the questionnaire independently, while illiterate participants were assisted by the investigator. The questionnaire was initially prepared in English and then translated into Amharic to ensure clarity and comprehension.

A sample of the administered questionnaire is provided as supporting information and referenced here for transparency and reproducibility.

#### 2.6.2. Collection and Examination of Blood Samples

From each participant, 5 mL of venous blood was collected from the median cubital vein in the forearm using standard aseptic phlebotomy procedures. The samples were placed into yellow‐top serum separator tubes, left to clot at room temperature, and then centrifuged at 3000 rpm for 5 min to isolate the serum. The separated serum was dispensed into prelabeled cryovials with unique participant identification codes and used for hepatitis serological analysis.

Serological testing for HBV and HCV infections was performed in the Debark General Hospital laboratory by medical laboratory personnel who underwent 2 days of training on phlebotomy techniques, biosafety, infection control, and study‐specific procedures for hepatitis B and C testing. HBV infection was identified through the detection of hepatitis B surface antigen (HBsAg), while HCV infection was determined by detecting anti‐HCV antibodies using commercially available rapid immunochromatographic assay kits (e.g., insert manufacturer, country). These tests rely on antigen–antibody reactions and lateral‐flow immunochromatographic technology, in which specific HBV antigens or HCV antibodies present in the patient sample bind to conjugated antibodies/antigens fixed on the test strip, leading to the appearance of a visible line.

Test results were interpreted strictly following the manufacturer′s guidelines. A sample was considered positive if both the control line and the test line were clearly visible. A negative result was defined by the presence of a distinct control line with no visible test line. Any test that lacked a control line or showed unclear or indeterminate test bands was classified as invalid; in these cases, the analysis was repeated using a new test cassette and a fresh serum aliquot.

### 2.7. Data Analysis

Data were verified for completeness, systematically coded, and entered into SPSS Version 23 for statistical analysis. Descriptive statistics, including absolute and relative frequencies (percentages), were calculated to characterize sociodemographic variables and seroprevalence. Univariate and multivariate logistic regression were applied to assess the relationships between potential risk factors and HBV‐HCV infection status. Variables with a *p* *v* *a* *l* *u* *e* ≤ 0.25 in the univariate analysis were subsequently entered into multivariate logistic regression models to determine independent predictors of infection. Statistical significance was defined as a two‐sided *p* *v* *a* *l* *u* *e* < 0.05, and the effect sizes were presented with their corresponding 95% confidence intervals (CIs).

## 3. Result

### 3.1. Sociodemographic Characteristics of Study Participants

A total of 256 pregnant women participated in the study, with a 100% response rate. The majority of participants were aged 20–29 years (53.5%), and the least represented age group was ≥ 40 years (2.34%). Most participants resided in urban areas (79.3%), whereas only 20.7% lived in rural areas. Regarding education, the highest proportion had completed secondary school (36.7%), and the lowest proportion was illiterate (9.76%). Most women were married (86.3%), while the fewest were divorced (2.73%). In terms of occupation, the majority were housewives (68.4%), and the least represented were employed in the private sector (12.5%) (Table 1).

**Table 1:** Sociodemographic characteristics of pregnant women attending antenatal care at Debark General Hospital from January to June 2024 (*n* = 256).

Variables		Frequency	Percentage (%)
Age (years)	< 20	21	8.2
	20–29	137	53.5
	30–39	92	35.9
	≥ 40	6	2.34
Residence	Rural	53	20.7
	Urban	203	79.3
Educational level	Illiterate	25	9.76
	Primary school	81	31.6
	Secondary school	94	36.7
	College and above	56	21.9
Marital status	Single	19	7.42
	Married	221	86.3
	Widowed	9	3.51
	Divorced	7	2.73
Occupation	Housewife	175	68.4
	Private	32	12.5
	Gov. employed	49	19.1

### 3.2. Seroprevalence of HBV and HCV

**Figure 1:**
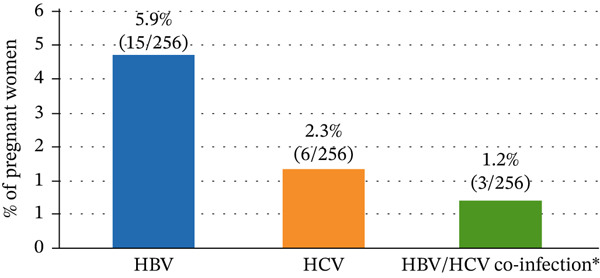
Seroprevalence of hepatitis B virus (HBV), hepatitis C virus (HCV), and HBV‐HCV coinfection among the study participants (*n* = 256).

The overall seroprevalence of HBV infection among the study participants was 5.9% (15/256), while HCV infection accounted for 2.3% (6/256). Concomitant HBV‐HCV coinfection was detected in 1.2% (3/256) of the participants. The distribution of HBV, HCV, and HBV‐HCV coinfection is illustrated in Figure 1.

### 3.3. Risk Factors Associated With the Prevalence of HBV Infection

Multivariate logistic regression revealed that sharp object injuries, previous contact with individuals infected with the HBV, and a history of blood transfusions were statistically significant predictors of HBV infection. Women who had experienced sharp object injuries had 3.5 times higher adjusted odds of testing positive for HBV (adjusted odds ratio [AOR]: 3.54; 95% CI: 1.54–7.20). Those who reported previous contact with HBV‐infected persons showed 6.3 times higher adjusted odds of HBV infection (AOR: 6.32; 95% CI: 2.20–12.80), and participants with a history of blood transfusion had 8.2 times higher adjusted odds (AOR: 8.20; 95% CI: 2.10–16.80). On the contrary, other variables evaluated, such as having multiple sexual partners, presence of body tattoos, ear piercing, tooth extraction, history of abortion, and pregnancy‐related complications, were not significantly associated with HBV infection in the adjusted analysis (Table 2).

**Table 2:** Univariate and multivariate analyses of risk factors potentially associated with HBV among pregnant women attending antenatal care at Debark General Hospital, 2024.

Variables		HBV positive (*n*, %)	HBV negative (*n*, %)	COR (CI)	*p* value	AOR	*p* value
Sharp object injury	Yes	11 (12.6%)	76 (87.4%)	6.22 (3.2–15.2)	≤ 0.001	3.5 (1.54–7.2)	**0.013**
	No	4 (2.37%)	165 (97.6%)	1		1	
Multiple sexual partners	Yes	12 (16.7%)	60 (83.3%)	3.8 (2.5–11.6)	0.01	1.56 (0.9–5.8)	0.066
	No	3 (1.63%)	181 (98.4%)	1		1	
Body tattooing	Yes	10 (10.4%)	86 (89.6%)	1.11 (1.23–3.6)	0.78		
	No	5 (3.12%)	155 (96.9%)	1			
Ear piercing	Yes	9 (6.0%)	141 (94.0%)	0.89 (0.99–3.4)	0.32		
	No	6 (5.66%)	100 (94.3%)	1			
History of contact with an HBV‐infected person	Yes	12 (19.4%)	50 (80.6%)	4.01 (1.42–11.4)	≤ 0.001	6.3 (2.2–12.8)	**≤ 0.001**
	No	3 (1.54%)	191 (98.4%)	1		1	
Tooth extraction	Yes	6 (7.79%)	71 (92.2%)	0.73 (0.8–4.3)	0.27		
	No	9 (5.03%)	170 (94.9%)	1			
Blood transfusion	Yes	8 (18.2%)	36 (81.8%)	13.62 (5.2–28.3)	≤ 0.001	8.2 (2.1–16.8)	**≤ 0.001**
	No	7 (3.30%)	205 (96.7%)	1		1	
Sexual partner′s HBV exposure	Yes	11 (37.9%)	18 (62.1%)	2.41 (1.21–12.4)	0.89		
	No	4 (1.76%)	223 (98.2%)	1			
Pregnancy‐related problems	Yes	7 (11.1%)	56 (88.8%)	1.43 (0.25–4.9)	0.26		
	No	8 (4.14%)	185 (95.8%)	1			
Abortion	Yes	4 (14.8%)	23 (85.2%)	6.13 (3.8–16.5)	≤ 0.001	1.2 (0.6–6.3)	0.054
	No	11 (4.8%)	218 (95.2%)	1		1	

*Note:* Bold *p* values indicate statistical significance (*p* < 0.05).

Abbreviations: AOR, adjusted odds ratio; COR, crude odds ratio.

### 3.4. Risk Factors Associated With the Prevalence of HCV Infection

Sharp object injuries, having more than one sexual partner, and a history of blood transfusion were significantly associated with HCV infection. Women who experienced sharp object–related injuries had 3.8 times higher odds of HCV infection (AOR: 3.8; 95% CI: 0.78–5.2). Those who reported having multiple sexual partners had 5.3 times higher odds (AOR: 5.3; 95% CI: 1.1–15.2), and women with a history of blood transfusion had 5.1 times higher odds (AOR: 5.1; 95% CI: 1.05–22.5). Additional variables were not identified as statistically significant predictors of HCV infection (Table 3).

**Table 3:** Univariate and multivariate analyses of HCV infection determinants among pregnant women attending antenatal care at Debark General Hospital from January 2024 to June 2024.

Variables		HCV positive (*n*, %)	HCV negative (*n*, %)	COR (95% CI)	*p* value	AOR (CI)	*p* value
Sharp object injury	Yes	2 (2.40%)	81 (97.6%)	5.3 (1.56–9.89)	0.009	3.8 (0.78–5.2)	**0.021**
	No	4 (2.31%)	169 (97.7%)	1		1	
Multiple sexual partners	Yes	4 (5.47%)	69 (94.5%)	6.3 (1.56–18.9)	0.01	5.3 (1.1–15.2)	**0.011**
	No	2 (1.09%)	181 (98.9%)	1			
Body tattooing	Yes	3 (2.68%)	109 (97.3%)	0.8 (0.8–5.81)	0.054	0.1 (0.09–2.5)	0.43
	No	3 (2.42%)	121 (97.6%)	1			
Ear piercing	Yes	4 (3.96%)	97 (96.0%)	1.8 (0.9–9.8)	0.263	2.8 (1.1–12.5)	0.06
	No	2 (1.29%)	153 (98.7%)	1		1	
History of contact with a hepatitis‐infected person	Yes	4 (6.89%)	54 (93.1%)	—	—	—	—
	No	2 (1.01%)	196 (98.9%)	—		—	—
Tooth extraction	Yes	2 (2.63%)	74 (97.4%)	1.1 (1.2–10.3)	0.09	1.5 (0.12–8.5)	0.52
	No	4 (2.22%)	176 (92.9%)	1		1	
Blood transfusion	Yes	2 (7.14%)	26 (92.9%)	6.2 (0.8–14.8)	0.03	5.1 (1.05–24.3)	**0.043**
	No	5 (2.19%)	223 (97.8%)	1		1	

*Note:* Bold *p* values indicate statistical significance (*p* < 0.05).

Abbreviations: AOR, adjusted odds ratio; COR, crude odds ratio.

### 3.5. Determinants of HBV‐HCV Coinfection

Multiple sexual partnerships and a history of contact with infected individuals were independently linked to HBV‐HCV coinfection. Women who reported having multiple sexual partners had 2.6 times higher odds of coinfection (*A* *O* *R* = 2.6; 95% CI: 1.2–12.8), while those who had previously been in contact with infected persons had 2.9 times higher odds (*A* *O* *R* = 2.9; 95% CI: 1.02–25.3). None of the other covariates evaluated showed a statistically significant association with HBV‐HCV coinfection (Table 4).

**Table 4:** Univariate and multivariate analyses of determinants of HBV‐HCV coinfection among pregnant women from January to June 2024.

Variables		HBV‐HCV coinfection positive (*n*, %)	HBV‐HCV coinfection negative (*n*, %)	COR (95% CI)	*p* value	AOR (CI)	*p* value
Sharp object injury	Yes	1 (2.27%)	43 (97.7%)	2.21 (0.06–11.21)	0.23	0.24 (0.02–13.3)	0.96
	No	2 (0.94%)	210 (99.1%)	1		1	
Multiple sexual partners	Yes	2 (7.14%)	26 (92.9%)	4.2 (0.21–13.5)	0.074	2.6 (1.2–12.8)	**0.02**
	No	1 (0.44%)	227 (99.6%)	1		1	
History of contact with an HCV‐HBV‐infected person	Yes	1 (4.98%)	19 (95%)	5.3 (0.3–12.5)	0.23	2.9 (1.02–25.3)	**0.01**
	No	2 (0.85%)	234 (99.2%)	1		1	
Blood transfusion	Yes	1 (4.55%)	21 (95.5%)	3.8 (0.21–8.34)	0.021	1.8 (0.25–6.85)	0.063
	No	2 (0.85%)	232 (99.2%)	1		1	
Abortion	Yes	2 (7.40%)	25 (92.6%)	6.8 (0.85–23.2)	0.089	7.9 (0.42–25.3)	0.09
	No	1 (0.44%)	228 (99.6%)	1		1	

*Note:* Bold *p* values indicate statistical significance (*p* < 0.05).

Abbreviations: AOR, adjusted odds ratio; COR, crude odds ratio.

## 4. Discussion

Viral hepatitis, primarily caused by the HBV and the HCV, remains a significant global public health concern, with a particularly high impact on pregnant women. In this group, infection poses a twofold threat: it can trigger the onset or worsening of liver disease in the mother and can result in vertical (mother‐to‐child) transmission. Perinatal transmission may give rise to chronic infection in the child, markedly increasing the lifetime likelihood of cirrhosis, hepatic decompensation, and hepatocellular carcinoma. This study assessed the seroprevalence and related determinants of HBV and HCV infections among pregnant women receiving antenatal care at Debark General Hospital to characterize local epidemiological trends and pinpoint relevant risk factors.

The total seroprevalence of HBV identified in this study was 5.9%, which corresponds to an intermediate level of endemicity. This result aligns with findings from northwest Ethiopia, including Gondar, where the prevalence of HBV infection among pregnant women has been reported to vary between 4% and 7% [17, 18]. The seroprevalence observed here is lower than that reported in Yemen (10.8%), Nigeria (9.3%), and Uganda (11.8%) but higher than that found in the Dawuro Zone (3.5%) and in several Middle Eastern countries, including Iran (0.7%) and Egypt (1.75%) [19]. These regional variations in the prevalence of HBV are likely influenced by differences in the coverage of the hepatitis B vaccine, routine antenatal screening programs, the availability and accessibility of healthcare services, and cultural and behavioral factors, as well as the types of diagnostic tests and case definitions used.

The seroprevalence of HCV in this study was 2.3%, which is similar to reports from the Gondar Hospital blood bank (2.33%), but lower than levels observed in Felege Hiwot Referral Hospital (13.3%) and Dessie Referral Hospital (6.5%) [20]. The comparatively lower prevalence in Debark may be due to a smaller occurrence of high‐risk behaviors, such as unsafe injections, blood transfusions, and other percutaneous exposures, as well as differences in demographic and clinical characteristics of the studied populations. Notably, Nkhata et al. [6] similarly reported that HCV infection was uncommon among pregnant women in Malawi, reinforcing the notion that HCV remains less prevalent than HBV in many sub‐Saharan African antenatal populations.

Coinfection with HBV and HCV was identified in 1.2% of the study population, a rate lower than that documented in Addis Ababa and Bahir Dar [19]. Although relatively uncommon, this coinfection has significant clinical implications, as it can accelerate the development of chronic liver disease and make therapeutic choices and overall patient care more complex, underscoring the need for accurate and robust diagnostic methods.

Analysis of possible risk factors revealed that sharp object injuries, previous contact with individuals infected with the HBV, and a history of blood transfusions were all significantly linked to HBV infection. Women who sustained injuries from sharp objects had 3.5 times higher odds of HBV infection, those who had previously come into contact with HBV‐infected persons had six times higher odds, and women who had ever received a blood transfusion faced a nearly eight‐fold increase in risk. These findings are consistent with previous research from Ethiopia, which identified percutaneous exposure and unsafe medical procedures as major pathways for HBV transmission [18, 21]. They also reflect trends reported by Nkhata et al. [6], who observed similar associations between percutaneous exposures and HBV infection in the Malawian antenatal population.

For HCV infection, key predictors included sharp objects, having multiple sexual partners, and a history of blood transfusion. Women who experienced sharp object injury had 3.8 times higher odds of infection, those with multiple sexual partners showed a 5.3‐fold increase in risk, and a history of blood transfusion was associated with a nearly five‐fold increase in the odds of HCV infection. Together, these results highlight the critical role of the percutaneous and sexual pathways of exposure in HCV transmission, especially in settings with limited resources [20]. Again, Nkhata et al. [6] observed that HCV prevalence was low, with percutaneous exposure as the main determinant, further confirming these transmission pathways across similar sub‐Saharan populations.

In the setting of coinfection with HBV‐HCV, having multiple sexual partners and a history of contact with infected individuals were identified as independent predictors. Women who reported multiple sexual partners had 2.6 times higher odds of coinfection, while those with a documented history of contact with infected persons had 2.9 times higher odds. These results highlight the need to integrate both behavioral and clinical risk factors into prevention efforts delivered through antenatal care services.

The public health significance of these findings is considerable. Vertical transmission of HBV remains a major problem, as in the absence of adequate maternal prophylaxis, up to 90% of exposed newborns can develop chronic infection [19, 21]. Although mother‐to‐child transmission of the HCV is less common, it also poses a substantial risk of chronic disease. Incorporating combined HBV and HCV screening into routine antenatal services, along with measures such as expanding hepatitis B vaccination coverage, maintaining strict adherence to safe medical procedures and blood transfusion protocols, and providing targeted, evidence‐based health education, is crucial to reducing morbidity among mothers and babies. The findings from Nkhata et al. [6] reinforce the relevance of these interventions in comparable sub‐Saharan African settings.

## 5. Limitations of the Study

This study has several limitations. First, its cross‐sectional nature prevents strong causal conclusions, limiting the findings to associations rather than temporal or causal links. Second, the sample size may have been insufficient to detect relationships involving less common risk factors, thereby reducing the statistical power. Third, certain exposures, especially those related to sexual practices, may have been underreported due to bias in social desirability, which can lead to misclassification of exposures. In addition, the laboratory methods used may not have detected all subclinical infections, which could have led to an underestimate of the actual prevalence. Despite these limitations, the study adds valuable evidence on the epidemiological burden of HBV and HCV among pregnant women and offers insights to guide interventions aimed at reducing both vertical and horizontal transmission.

## 6. Conclusions and Recommendations

The study showed that HBV and HCV remain the main public health problems among pregnant women at Debark General Hospital, with seroprevalence rates of 5.9% and 2.3% and a coinfection rate of HBV‐HCV of 1.2%. HBV infection was significantly associated with sharp object injury, contact with known infected individuals, and prior blood transfusion. HCV infection was significantly associated with sharp object injuries, multiple sexual partners, and prior blood transfusion. Coinfection was independently associated with multiple sexual partners and contact with infected persons. These findings underscore the risk of mother‐to‐child transmission and the need for integrated prevention. Routine detection of HBV and HCV should be part of antenatal care, HBV vaccination, and neonatal prophylaxis should be strengthened, and adherence to safe clinical and infection prevention practices should be improved. Additionally, targeted health education on behavioral risk factors is needed. Together, these measures could reduce the burden of viral hepatitis among pregnant women and improve maternal and newborn outcomes, especially in settings with intermediate endemicity.

## Author Contributions

G.A.A. was responsible for the conceptualization and design of the study, performed data extraction and analysis, interpreted the results, and drafted the manuscript. S.T. contributed to the selection of studies, quality assessment, interpretation of the results, and review of the manuscript.

## Funding

No funding was received for this manuscript.

## Ethics Statement

The study protocol was approved by the Ethics Committee of Debark University (Ref. No.: REC/DKU/046/2024), and institutional authorization was obtained from Debark General Hospital. All participants were informed about the purpose, procedures, and possible risks and benefits of the study, and each provided their written informed consent before collecting blood samples. Each pregnant woman received a unique identification code, and personal identification codes were accessible only to the designated research team, ensuring confidentiality. Participation was voluntary, and participants were informed that they could withdraw at any time, before or after stool sample collection, without loss of hospital benefits.

## Consent

Written informed consent was obtained from pregnant women who participated in the study. They were informed about the study and agreed to participate voluntarily.

## Conflicts of Interest

The authors declare no conflicts of interest.

## Supporting Information

▪ Sociodemographic information (age, residence, education, marital status, and occupation)
▪ Obstetric and medical history (pregnancy‐related problems, abortion, blood transfusion, and tooth extraction)
▪ Behavioral and exposure risk factors (sharp object injuries, multiple sexual partners, tattoos, ear piercings, and contact with hepatitis B or C–infected individuals)

## Supporting information


**Supporting Information** Additional supporting information can be found online in the Supporting Information section. Supporting Information.docx contains the questionnaire used to collect data from pregnant women attending antenatal care at Debark General Hospital. The questionnaire includes sections on.

## Data Availability

The datasets used and/or analyzed during the current study are available from the corresponding author upon reasonable request.
