# Supplementary material for: Seroprevalence and Determinants of Hepatitis B and C Viral Infections Among Pregnant Women Attending Debark General Hospital, Northwest Ethiopia
Source: Biomed Res Int. 2026 Feb 23;2026:6510690. doi: 10.1155/bmri/6510690 (PMC12929633; doi:10.1155/bmri/6510690)
Supplement: Supplementary file 1 — Supporting Information Additional supporting information can be found online in the Supporting Information section. Supporting Information.docx contains the questionnaire used to collect data from pregnant women attending antenatal care at Debark General Hospital. The questionnaire includes sections on. [file BMRI-2026-6510690-s001.docx]

**Supplementary File**

**Questionnaire for Pregnant Women Attending Antenatal Care at Debark General Hospital**

**Section A: Sociodemographic Information**

1. Age: ___ years
2. Residence: File forhepatitis☐ Urban ☐ Rural
3. Educational Level: ☐ Illiterate ☐ Primary school ☐ Secondary school ☐ College and above
4. Marital Status: ☐ Single ☐ Married ☐ Widowed ☐ Divorced
5. Occupation: ☐ Housewife ☐ Private sector ☐ Government employee ☐ Other: ______

**Section B: Obstetric and Medical History**
6. Have you ever experienced pregnancy-related problems? ☐ Yes ☐ No
7. Have you ever had an abortion? ☐ Yes ☐ No
8. Have you ever had a blood transfusion? ☐ Yes ☐ No
9. Have you ever had a tooth extraction? ☐ Yes ☐ No

**Section C: Behavioral and Exposure Risk Factors**
10. Have you ever sustained a sharp object injury (e.g., needle stick, cut)? ☐ Yes ☐ No
11. Have you ever had multiple sexual partners? ☐ Yes ☐ No
12. Do you have a body tattoo? ☐ Yes ☐ No
13. Do you have ear piercings? ☐ Yes ☐ No
14. Has your sexual partner ever been diagnosed with hepatitis B or C? ☐ Yes ☐ No ☐ Don’t know
15. Have you ever had close contact with a person known to be infected with hepatitis B or C? ☐ Yes ☐ No ☐ Don’t know
